# Non-Absorbing Dielectric Materials for Surface-Enhanced Spectroscopies and Chiral Sensing in the UV

**DOI:** 10.3390/nano10102078

**Published:** 2020-10-21

**Authors:** Saúl A. Rosales, Francisco González, Fernando Moreno, Yael Gutiérrez

**Affiliations:** 1 Department of Applied Physics, University of Cantabria, Avda. Los Castros, s/n., 39005 Santander, Spain; rosalessa@unican.es (S.A.R.); gonzaleff@unican.es (F.G.); 2 Institute of Nanotechnology, CNR-NANOTEC, Via Orabona 4, 70126 Bari, Italy

**Keywords:** dielectric nanoparticles, chirality, chiral molecules, surface-enhanced spectroscopies, Mie resonances, UV

## Abstract

Low-loss dielectric nanomaterials are being extensively studied as novel platforms for enhanced light-matter interactions. Dielectric materials are more versatile than metals when nanostructured as they are able to generate simultaneously electric- and magnetic-type resonances. This unique property gives rise to a wide gamut of new phenomena not observed in metal nanostructures such as directional scattering conditions or enhanced optical chirality density. Traditionally studied dielectrics such as Si, Ge or GaP have an operating range constrained to the infrared and/or the visible range. Tuning their resonances up to the UV, where many biological samples of interest exhibit their absorption bands, is not possible due to their increased optical losses via heat generation. Herein, we report a quantitative survey on the UV optical performance of 20 different dielectric nanostructured materials for UV surface light-matter interaction based applications. The near-field intensity and optical chirality density averaged over the surface of the nanoparticles together with the heat generation are studied as figures of merit for this comparative analysis.

## 1. Introduction

Nanophotonic devices have been intensively investigated aiming the development of new spectroscopic techniques such as surface-enhanced Raman spectroscopy (SERS) [1,2,3,4], surface-enhanced infrared absorption (SEIRA) [4,5,6,7], surface-enhanced fluorescence (SEF) [2,8,9,10,11] or chiral sensing [12,13,14], among others. Generally, these spectroscopic techniques rely on the hot-spots produced when nanostructures are illuminated under resonance. A molecule located in those hot spots will exhibit an enhanced interaction with light that produces measurable changes in the scattered light properties. The stronger the interaction, the larger the resulting modifications in the light properties. Consequently, the sensitivity to the presence of molecules is increased. To achieve such strong interactions, two key factors must be taken into account. The first one is related to the amplitude (intensity and polarization) of the electromagnetic field, which has to be optimized to efficiently induce optical transitions in the target molecule. In most cases of SERS, SEIRA and SEF only enhanced electromagnetic field intensities, commonly named as near-field enhancement (NFE), at the surroundings of the molecule are required [1,5]. Therefore, the NFE on the nanostructure’s surface is the main figure of merit for comparing different nanomaterials applied to the aforementioned spectroscopy techniques. However, in some other cases, due to the symmetry properties of the molecule, some electronic transitions impose selection rules over light polarization. A clear example of this are molecules showing chiral properties [15]. A molecule is chiral when it cannot be superimposed to its specular image. The pair of non superimposable image molecules are commonly known as enantiomers, each of them referred as left- and right-handed. Due to chirality, each enantiomer absorbs differently left and right circularly polarized light, i.e., they exhibit circular dichroism (CD) [12,15]. The CD that chiral molecules exhibit is proportional to the light’s optical chirality density (OCD) of the electromagnetic field in the surroundings of the molecule. For this reason, the OCD on the nanostructure’s surface is the figure of merit when comparing nanomaterials for chiral sensing purposes. This quantity depends not only on the electric and magnetic fields intensity but also on their polarization properties [16]. We will discuss OCD later on.

**Figure 1:**
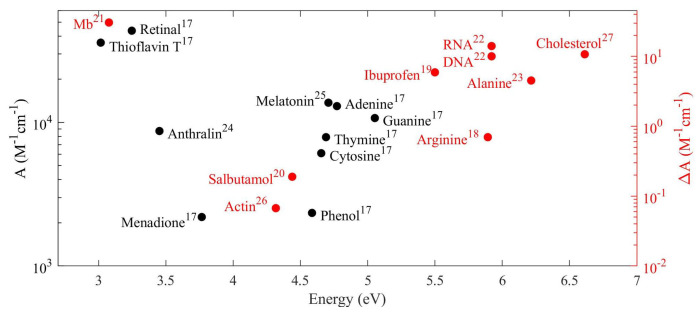
Organic molecules absorption bands: (left axis) molar absorption extinction, *A*, for some non-chiral molecules; (right axis) difference between molar absorption extinction of left- and right-circularly polarized light, ΔA , for some chiral molecules.

The second key factor consists of resonantly matching the exciting light frequency with the molecule’s natural frequency. This condition is essential for a good performance in spectroscopic techniques based in molecular absorption. Most molecules have their molecular vibrational and rotational frequencies in the infrared spectrum [1] while the electronic transitions fall in the visible and UV range. For example, many organic compounds such as small pharmaceuticals and biomolecules have electronic transitions at energies in the UV range [17,18,19,20,21,22,23,24,25,26,27], as shown in Figure 1. Therefore, to achieve optimal frequency matching between hot-spots and molecule’s electronic transitions, the selected nanomaterials must have maximum optical response in the UV range.

Metal-based nanostructures enhance near-fields intensity with inherent heating of the structure and its surroundings due to their high resistive losses [2,28,29,30]. Therefore, they are interesting for applications where heating is desired or at least this is not an issue [29,30,31]. It is important to note that heating effects are detrimental in many ways for spectroscopic measurements. The temperatures generated when the plasmonic resonances are excited can reach up to several hundreds of degrees Kelvin [2,31,32,33]. These temperatures are high enough to generate substantial changes in the nanomaterial and environment such as deformation or even melting of nanostructures, vaporizing the solvent, refractive index changes through the thermo-optic effect and molecular changes in the samples [32,33]. All these changes contribute to the spectral shift of both the plasmonic and molecular resonances complicating the data analysis and diminishing sensing efficiencies. So, not only in the UV but also in the infrared and visible range, alternative materials to metals are demanded for spectroscopic measurements.

Very recently, semiconductors have been proposed for infrared and visible photonic applications with ultralow heat radiation [2,3,6,9,10,14,34,35,36,37,38]. Especial interest is being given to semiconductor materials with high refractive index (HRI) and low losses. These materials, when nanostructured, are able to generate high NFE with negligible heating [2] through the excitation of both, electric and magnetic resonances. Under appropriate nanostructuring and illumination conditions, a maximum interference between this two type of resonances can be achieved, leading to attractive phenomena like directional scattering [39,40] or OCD hot spots [13,41,42,43].

Metals for UV applications have been already thoroughly reviewed in terms of absorption and NFE [28,29,44]. Some HRI materials ( n≥2 ) have been already discussed in terms of directionality and NFE with low heat conversion in the UV showing as promising candidates diamond or TiO_2_ among others [45]. In general terms, insulators and semiconductors with a sufficiently wide band gap ( $E_{g}\geq 3$ eV) have a transparency window in the UV. However, such wide band gaps imply a relatively low refractive index when compared to narrow band gap semiconductors [46]. Therefore, not only HRI, but also lower refractive index materials should be considered to obtain NFE with negligible heating effects in the UV.

The purpose of this research is intended to show the performance of 19 potential low-loss dielectric materials. We show that this materials, when nanostructured, generate surface light-matter interaction enhancements based in both the NFE and OCD response in the UV range. This enhancements are relevant for UV sensing applications, especially those related to the bio-field since in this spectral range many biological molecules have absorption bands. In addition, our aim is to continue with the recent research on chiral sensing in the UV with low-loss HRI dielectrics [47]. To do so we analyze the optical chirality performance of the proposed materials in the UV range. We envision that this study can open a new landscape for optimizing the design and the efficiency of new surface-interaction based sensing platforms with alternative low loss dielectric materials avoiding the sometimes harmful heating. Materials with HRI, n≥2 , moderate (MRI), 1.7≤n<2 , and low refractive index (LRI), n<1.7 are considered and compared. NFE and OCD calculations, based on Mie theory [48,49], are done for all the materials proposed [50,51,52,53,54,55,56,57,58,59,60,61,62,63,64,65]. To the best of our knowledge, these materials have not been proposed yet as promising UV nano-alternative for surface enhanced spectroscopy. The aforementioned calculations for diamond [66] are also included for comparison since it has been previously proposed for VIS/UV-HRI photonics [45,47,67,68].

This manuscript is organized as follows. In Section 2, we briefly review the theoretical concepts and methods used to perform the electromagnetic calculations. In Section 3, we describe and classify the list of materials under review. In Section 4, we compare their optical response in the UV. This comparison is done in terms of NFE and OCD resonances, considering also relevant features of the resonances, such as absorption efficiency and width. Finally in Section 5, we remark the main findings of this work and summarize its main conclusions.

## 2. Methods

### 2.1. Mie Theory

A nanosphere shares many common features with nanoparticles of arbitrary shape, such as resonant scattering at different wavelengths, electric and magnetic type resonant field patterns, geometrical tunability of resonances, etc. One important difference for the spherical geometry is that the electromagnetic solution is analytical. This allows fast computations of electromagnetic fields with only a few variables to consider while keeping the physics background of the problem. For most spectroscopic applications, the fundamental magnitudes to consider are the electromagnetic fields in the close proximity to the particle, i.e., those fields acting on a possible target molecule. In the case of a spherical particle, the scattered electric and magnetic fields ( $E_{s}$ and $H_{s}$ ) can be expressed as multipole series [48,49],

(1) $$E_{s}=E_{0}\sum_{n=1}^{\infty }\frac{i^{n}\left(2n+1\right)}{n(n+1)}\left[ia_{n}\left(u_{x}N_{e1n}^{\left(3\right)}+u_{y}N_{o1n}^{\left(3\right)}\right)-b_{n}\left(u_{x}M_{o1n}^{\left(3\right)}-u_{y}M_{e1n}^{\left(3\right)}\right)\right]$$

(2) $$B_{s}=\frac{kE_{0}}{\omega }\sum_{n=1}^{\infty }\frac{i^{n}\left(2n+1\right)}{n(n+1)}\left[ib_{n}\left(u_{x}N_{o1n}^{\left(3\right)}-u_{y}N_{e1n}^{\left(3\right)}\right)+a_{n}\left(u_{x}M_{e1n}^{\left(3\right)}+u_{y}M_{o1n}^{\left(3\right)}\right)\right]$$

where $N_{e1n}^{\left(3\right)}$ , $N_{o1n}^{\left(3\right)}$ , $M_{o1n}^{\left(3\right)}$ and $M_{e1n}^{\left(3\right)}$ are the vector spherical harmonics as defined in [48], ω and *k* are the angular frequency and wave vector of the incident field, and μ is the permeability of the surrounding medium. $a_{n}$ and $b_{n}$ are the Mie scattering coefficients. From a physical point of view, those coefficients represent the weighting factors of the different electric and magnetic multipolar contributions of order *n* (e.g., n=1 dipolar, n=2 quadrupolar). These coefficients depend on the permitivitty and permeability of the sphere and those of its surroundings, the sphere’s size and the frequency of the electromagnetic field. The exciting plane wave is defined by its electric field amplitude, $E_{0}$ , and by its normalized transverse components $u_{x}$ and $u_{y}$ , assuming it propagates in the z-direction.

### 2.2. Optical Chirality

The energy absorption rate from an electromagnetic field by a chiral molecule is [16],

(3) $$A^{\pm }=\frac{\omega }{2}(\alpha^{\prime \prime }{\left|E\right|}^{2}+\chi^{\prime \prime }{\left|B\right|}^{2})\pm G^{\prime \prime }C$$

$\alpha^{\prime \prime }$ and $\chi^{\prime \prime }$ are the imaginary parts of the electric polarizability and magnetic susceptibility of the chiral molecule, respectively. $G^{\prime \prime }$ is the imaginary part of the electric-magnetic dipole polarizability, assumed to be isotropic, and responsible of the chiral character of the molecule. Finally, ω and *C* are the angular frequency and the OCD, respectively, of the electromagnetic field acting on the molecule. *C* is given by [16]

(4) $$C=-\frac{\omega }{2c^{2}}𝔍\left(E^{*}\cdot B\right)$$

where *c* is the speed of light in vacuum, and E and B are the electric and magnetic fields acting on the molecule. It is worth noting that when considering molecules in the vicinity of a NP, these fields always consist on the superposition of the incident and the scattered fields.

In chiral sensing experiments, the discrimination between left and right handed molecules arises exclusively from the absorption due to the material chirality $G^{\prime \prime }$ and the OCD of the electromagnetic wave, *C*. By contrast, the first absorption term in Equation (3) is equal for left- and right-handed molecules. Thus, for enantiomer separation, it is desirable an absorption dominated by the chirality term. This feature is taken into account in the Kuhn’s dissymmetry factor [16],

(5) $$g=-\left(\frac{G^{\prime \prime }}{\alpha^{\prime \prime }}\right)\left(\frac{2C}{\omega U_{e}}\right)$$

where $U_{e}$ is the time-averaged electric energy density.

For a plane wave, the values of *C* and *g* are maximum for circularly polarized light (CPL) and their values are given by

(6) $$C_{CPL}=\pm 2\frac{\omega U_{e}}{c}$$

(7) $$g_{CPL}=\frac{-4G^{\prime \prime }}{c\alpha^{\prime \prime }}$$

It is well established that, in the near-field regime, many dielectric nanostructures enhance both the OCD and the Kuhn’s dissymmetry factor when compared to the plane wave values [13,16,47,69], i.e., $C/C_{CPL}>1$ and $g/g_{CPL}>1$ . This enhancement is due to the possibility of the simultaneous excitation of electric and magnetic modes allowed in dielectric nanostructures [41]. Of special interest is the OCD enhancement in the UV, i.e., the spectral range in which most of chiral molecules of industrial interest exhibit absorption bands as shown in Figure 1.

By using Equations (1) and (2) to calculate the electromagnetic fields, and Equation (4) to calculate the OCD generated in the nanosphere surroundings, we will evaluate the potential use of materials not considered before in applications related to surface enhanced spectroscopies and chiral sensing in the UV. This will be treated in the following sections.

## 3. Materials under Study

Here, we are mainly interested in materials that offer an acceptable transparency (i.e., low absorption) from 3 to 6 eV. The band gap energy gives an idea of the range of the transparency window for a given material. However, two materials with a similar bandgap energy may exhibit two very different absorption spectra. This difference is related to both the crystalline and electronic structure which strongly depends on the constituent elements of the material. At first glance, a material with a band gap superior to 3 eV is a promising candidate for UV applications. Many materials satisfy this condition. They can be categorized in four main groups attending to the constituent elements: oxides, nitrides, halides and chalcogenides. Here, 20 UV transparent materials, including representative materials of each of those groups are listed. Some more can be found, for example in Ref. [70].

**Figure 2:**
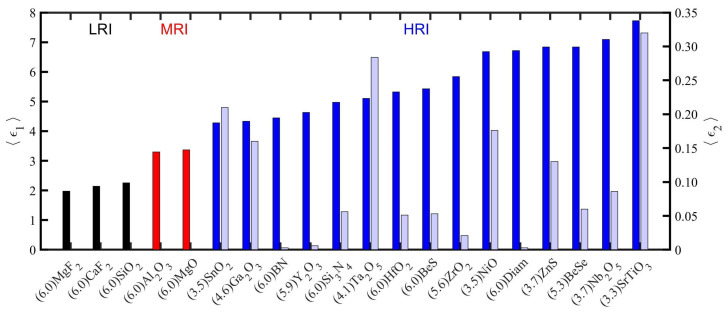
Mean real part (left axis) and mean imaginary part (right axis) of the permittivity calculated from 3 eV to the energy indicated between parenthesis below the compound label (x-axis) of all the 20 materials studied in this work.

For quick comparisons of optical constants without missing important information, a simple yet rigorous criterion is needed. For each material, we obtained from the Refs. [50,51,52,53,54,55,56,57,58,59,60,61,62,63,64,65,66] the energy for which the imaginary part of the complex dielectric function, $\epsilon_{2}$ , exceeds the value of 0.5. For example, the amplitude of a plane wave propagating through a dielectric media with $\epsilon_{2}=0.5$ and $\epsilon_{1}=4$ decays a factor 1/e for path-lengths roughly exceeding the wavelength. So, for this transparency conditions one can still expect some resonant response for subwavelength dielectric nanospheres (for more details see Supplementary Information, SI). Under this criterion, we differentiated two main groups of materials: those with $\epsilon_{2}>0.5$ in the region of interest (3 to 6 eV) and those with $\epsilon_{2}<0.5$ in part of the same region. To facilitate comparison between the two groups, Figure 2 shows average value of the real ( $\langle \epsilon_{1}\rangle$ , solid bars) and imaginary part ( $\langle \epsilon_{2}\rangle$ , shaded bars) of the permittivity of each of the studied materials. This average is calculated in a range that starts at 3 eV and end at the energy indicated below each compound formula (x-axis).

The first group of materials considered in this work present a strong absorption edge for energies below 6 eV. Conveniently, as an indicative of this issue, we show in Figure 2 the energy at which $\epsilon_{2}$ exceeds the 0.5 threshold along with the material’s label. From 3 eV to that energy, the mean value of $\langle \epsilon_{1}\rangle$ and $\langle \epsilon_{2}\rangle$ are also indicated. In that region, slight dispersion is observable, and the mean values of $\langle \epsilon_{2}\rangle$ takes values lower than 0.35 in all cases, and in most cases below 0.2. Therefore, the selected materials show low absorption in that part of the near UV spectrum. A detailed information of the optical constants is shown in the SI. The rest of the selected materials are transparent in the 3–6 eV spectral range under the criteria defined here. These materials have their direct band gaps above 6 eV, a $\epsilon_{1}$ with low dispersion and negligible $\epsilon_{2}$ . For these materials, in Figure 2, we also show the mean real and imaginary part of the permittivity calculated but in the range 3–6 eV. In the case of LRI and MRI materials, the imaginary part is so small that can be considered identically to zero.

Table 1 summarizes the materials analyzed in this research together with their primary applications or potential applications and reported nanostructures based on those materials. Semiconductors having a bandgap between 3 and 6 eV have a good transparency in the visible and part of the near UV spectral range. This semiconductors are often called wide bandgap semiconductors (WBS). If a semiconductor of these characteristics can be also crystallized with good quality, cheap costs and controllable doping, it becomes an interesting candidate for many electronic and optoelectronic applications. In Table 1 the following applications concerning electronics are listed: metal-oxide-semiconductor field effect transistor (MOSFET), ion-sensitive field effect transistor (ISFET), gas sensing (GS), dynamic random access memory (DRAM), magnetoresistive random access memory (MRAM) and resistive random access memory (RRAM). For all this devices, metals, semiconductors and insulators are simultaneously integrated in the micrometer and nanometer scale. Some of the materials listed here have been intensively studied as building material for the insulator and/or semiconductor parts of the devices, as it can be seen in the references indicated in Table 1. In some cases like BN, diamond and $Ga_{2}O_{3}$ , due to their thermal properties and/or electronic properties are suitable for the design of the aforementioned applications but for operating at high temperature (HT) and/or high power (high power devices, HPD). In addition, the materials here studied have been proposed for many optoelectronic applications like: luminiscent devices (LumD), dye synthesize solar cell (DSSC), Schottky barrier diode (SBD), visible blind UV photodetector (UV-PD) and transparent conductive film (TCF).

Finally, applications concerning photonics are also listed: bulk optical devices (BOD), fiber optics (FO), coating and substrates (CS), solid state laser (SS-LASER), photocatalysis (PCat), surface enhanced spectroscopy (SES) and optical quantum computing (OQC). The three last mentioned research fields are still in their early-stage state but each one of them have important reasons to end up being relevant topics in applied physics. For example, PCat is an interesting alternative to the inefficient thermal catalysis. The catalysis is crucial for industrial production of many pharmaceuticals and hydrogen which is one of the promising future clean energy sources. SES consist in the employment of light waveguides or resonators (micrometer or nanometer scale) for improving the detection limits of conventional light spectroscopy. This enables more reliable and efficient study of matter properties which has immediate applications in disease and/or contaminants detection. Quantum computers are the next generation of computers that relies on a inviolable security encoding of data, the quantum bits or Q-bits. This data encoding consist in storing the information, bits, in quantum states of a quantum systems (atoms, molecules, quantum light, etc...). So for this technology the precise manipulation of quantum states is needed (generation, transportation, lecture/measurement). Single photon emitters/detector joined with integrated photonic circuits opens a direct way of implementing quantum computing at room temperature [68] which is the main advantage of OQC against more developed quantum computer based in superconducting materials [146]. So one must notice that all the properties of the materials listed here, except BeS and BeSe due to toxicity issues, have been intensively studied in other fields but not yet for spectroscopic purposes, except SiO_2_ [78], Si3N_4_ [6], ZnS [141] and C(Diam) [47] in the infrared and/or the visible range. Mostly, the rest have been studied for other purposes as nanomaterials. As an indicative, in the third and fourth column of Table 1 some references reporting lithography and nanocrystal growing based on the materials listed here are shown.

## 4. Results

### 4.1. Near-Field Enhancement and Absorption Efficiency

The physical origin of resonances in metallic and dielectric nanoparticles is different [35,147,148]. For metals, the resonance arises from the presence of free electrons, a fact that is intimately related to the negative values of the real part of their permittivity. A plasma of free electrons may oscillate coupled with the exciting light leading to plasmonic resonances that may be excited for very small sized nanoparticles. In the case of metallic spheres, when they are small compared with the wavelength, the electric dipolar resonance, which is the lower order resonance, is excited at the energy where ϵ≈−2 , known as the Fröhlich energy [147,148]. For dielectrics, the origin of the resonance is a subtle combination of the optical properties, i.e., the refractive index of the particle $n_{in}$ , the surrounding refractive index $n_{ex}$ , and the nanoparticle geometrical size, e.g., the nanosphere radius *R*. In the latter case, the ratio $Rn_{in}/\left(n_{ex}\lambda \right)$ should be large enough so, whispering gallery modes may be excited. The magnetic dipolar resonance, which is the lower order resonance in dielectric nanospheres, is excited when $2\pi Rn_{in}/\left(n_{ex}\lambda \right)\approx$ 3 [34,35]. There are two magnitudes which can resonate in a nanoparticle and are very attractive for experimenters. One is the value of the electric field intensity, ${\left|E\right|}^{2}$ , close to the nanoparticle, and the other is its absorptive capacity of the incident radiation represented by the absorption efficiency, $Q_{abs}$ , which is given by the absorption cross-section divided by the geometrical cross section of the nanoparticle.

When achiral and isotropic samples are analyzed, the light absorption is only affected by the molecular polarizability and field intensity. So, in this case, a high field intensity is desired to enhance the molecular absorption and thus obtain more detailed matter information [149,150]. This is the basis of many surface-enhanced spectroscopic techniques like SERS [149,150], SEIRA [150], and SEF [150,151]. Because the electric near-field intensity, ${\left|E\right|}^{2}$ , varies with position in the nanoparticle surface, we average this magnitude over the sphere’s surface, denoting the average as $\langle |E{|}^{2}\rangle$ . To perform such an average, a set of equi-distributed points must be generated on the surface of the nanoparticles. For spherical geometries, this is not straightforward for an arbitrary number of points. Here, the set of points for evaluating the mean of ${\left|E\right|}^{2}$ is carried out by following the procedures explained in [152]. Of course, that average will be normalized to the incident electric field intensity $|E_{0}{|}^{2}$ . Apart from the peak values of $\langle |E{|}^{2}\rangle$ , the half-width at half-maximum (HWHM) of the $\langle |E{|}^{2}\rangle$ resonances and the absorption efficiency $Q_{abs}$ at the energy of the maximum $\langle |E{|}^{2}\rangle$ , are magnitudes to be considered. Large values of $Q_{abs}$ result in high absorption rates and thus a non-negligible heating of both the sample and nanostructure, which can result in detrimental changes in their properties [2]. To avoid this, low $Q_{abs}$ values are desirable in the $\langle |E{|}^{2}\rangle$ resonance energy. On the other hand, small HWHMs (high *Q*-factors) are desired for enhancing detection limits. For exploiting the best yield of high *Q*-factors resonance, an almost perfect tuning between the light source, the nanostructure resonance, and the molecule’s natural frequency is critical. This tuning is not always trivial to fulfill. Consequently, depending on the experimental conditions (light source, manufacturing precision, molecule’s absorption band, etc.), high *Q*-factors are important or unnecessary. In this section, we analyze these parameters and their relationship for the materials considered in this research.

**Figure 3:**
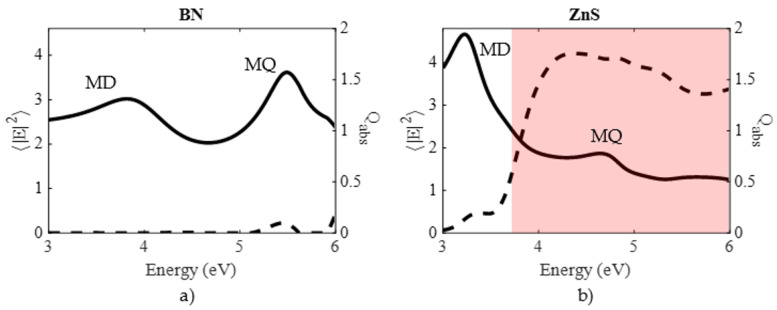
Surface-averaged electric field intensity (solid line, left axis) and absorption efficiency (dashed line, right axis) for (**a**) BN and (**b**) ZnS nanospheres of 70 nm radius. The red shaded area indicates the range where $\varepsilon_{2}>0.5$ .

As a first example, the typical spectral tendency of those magnitudes are shown in Figure 3. In this figure, the spectral dependence of $\langle |E{|}^{2}\rangle$ for a sphere with radius R=70 nm is shown for two representative materials: BN and ZnS. BN is a material that has a small $\epsilon_{2}$ with values lower than 0.05 from 3 to 6 eV. Contrarily, for ZnS, $\epsilon_{2}>0.5$ for energies above 3.5 eV, as indicated by the red shaded area. For both materials the absorption efficiency, $Q_{abs}$ , is also shown. In the case of BN, the values of the absorption efficiency are relatively small in the range 3–6 eV. This fact is related to its low values of $\epsilon_{2}$ . For ZnS, in the shaded area where $\epsilon_{2}$ is not negligible, $Q_{abs}$ has high values which are comparable to those reached in metals [28]. $Q_{abs}$ is related to the light absorption rate, and consequently to the heat generated by the nanostructure. Heat generation can have a critical role both in the sample and the nanostructures. On one hand, if the analyzed sample is sensitive to temperature changes, the spectroscopic measurements performed at photon energies with non negligible values of $Q_{abs}$ , will be affected. Moreover, for high temperatures the sample can be vaporized. On the other hand, an increase of the nanostructure temperature can induce damages such as melting and/or reshaping, and lead to changes in the refractive index via the thermo-optic effect [32,33]. One important aspect to note in the case of dielectric nanostructures, is that high values of $Q_{abs}$ implies low values of field enhancement as it can be seen in the ZnS spectrum. On the contrary, for metals this is not always true [28]. For example, a 70 nm radius Ag sphere, at the localized surface plasmon resonance (LSPR) energy (3.5 eV) generates a maximum surface averaged field enhancement of ≈25 with an absorption efficiency of ≈1.8.

**Figure 4:**
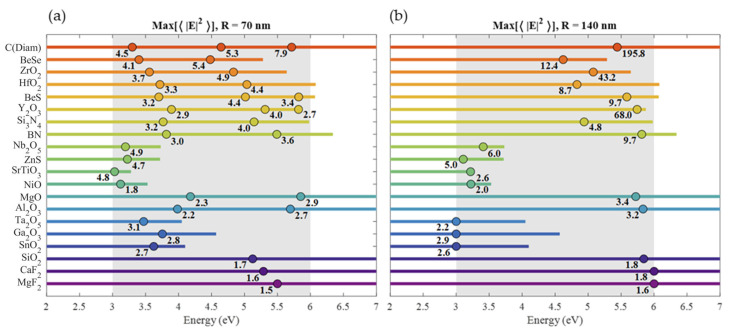
Summary of the $\langle |E{|}^{2}\rangle$ maxima values in the [3, 6] eV range for (**a**) 70 nm and (**b**) 140 nm radius spheres. For 70 nm radius, only magnetic resonances are indicated, while for 140 nm radius, only the most intense resonance is indicated. The dots indicate the energies of the maxima, while the line length indicates the range of energies where $\varepsilon_{2}<0.5$ . Under each dot, the maximum value of $\langle |E{|}^{2}\rangle$ is indicated.

For the comparison of all the HRI materials considered here, we have chosen two radii for the sphere particle model: R= 70 and 140 nm. In Figure 4, we compare the spectral maximum of $\langle |E{|}^{2}\rangle$ for each material (noted with its numerical value) while indicating with a point the energy where that maximum is reached (only resonances between 3 a 6 eV are considered). With an horizontal line, the spectral range where $\epsilon_{2}<0.5$ is also indicated. In this range, the presented resonances can be tuned by means of the sphere radius with slight changes in the maximum due to their small refractive index dispersion. This is another important difference when comparing dielectric with metallic nanostructures. For the latter, the plasmonic response strongly decays with increasing size [153]. In the case of R=70 nm, the position of all the magnetic resonances are shown. These resonances are more intense and narrow than the electric resonances (higher *Q*-factor). Materials with only one point exhibit only a magnetic dipole (MD) resonance, which in terms of the Mie series, corresponds to the coefficient $b_{1}$ . For the materials that exhibit two or more resonances, the maxima indicated correspond, from left to right, to the MD, magnetic quadrupole (MQ, $b_{2}$ ) and magnetic octopole (MO, $b_{3}$ ) resonances, respectively. It’s important to know that between the MD and MQ resonances there is always an electric dipole resonance (ED, $a_{1}$ ). However, this resonance is so wide that only appears as a background in the spectrum. Analogously, between the MQ and MO, an electric quadruple resonance (EQ, $a_{2}$ ) is often obtained, and this appears as a less intense and wider resonance than the MD, MQ and MO resonances. For this reason, and for simplicity, electric type resonances were obviated for the 70 nm radius case in Figure 4. In the case of R=140 nm spheres, the number of resonances becomes too large for some HRI materials, so only the most intense maximum is indicated. In addition, for each radius, the value of the absorption efficiency at the maximum of $\langle |E{|}^{2}\rangle$ and its HWHM are shown in Figure 4. For 70 nm radius spheres, these two magnitudes are obtained for the magnetic dipolar resonance (MD) since every material in the list support this resonance for that radius in the region of interest. For the R=140 nm radius, these magnitudes were obtained for the most intense resonance.

Resonances of 70 nm spheres are relatively weak when compared to values reached with some small metallic nanoparticles in the UV or in the visible range [28,153]. Nevertheless, the resonances shown in Figure 4 could be tuned, with small changes in the peak value to match the energy of molecular absorption bands energies where the polarizability is several orders of magnitude larger compared to the values in the visible range. Contrarily, the extreme high values reached in metallic nanospheres only occurs for specific values of the complex permitivitty, and consequently, at specific energies. For example, as it was studied in [153], the ED resonance exhibited its best response when the sphere radius was of a few nms, at the Fröhlich energy, that was fixed for each metal. When the resonance was tuned away from that energy by increasing the sphere radius, the peak values of the NFE decreased rapidly and end up being comparable to the ones reported here for dielectric spheres. Analogously, the same occurred for the QE resonance in metals which exhibited its best response at the energy where ϵ≈−1.5 [153], for radius of tens of nm. This issue, together with the unavoidable high $Q_{abs}$ values in LSPR, makes dielectric nanomaterials a very interesting alternative to metals. In addition, it is important to note that extremely high values of NFE could also be reached with HRI materials if the sphere was big enough. For example, the R=140 nm diamond sphere showed NFE peak values larger than 300 in the spectral region of interest with negligible heating effects ( $Q_{abs}<<10^{-4}$ ). However, the HWHM of these resonances dropped to values lower than 1 meV. This makes it challenging to achieve a tuning between the light and nanoresonator resonant energy. In other words, the manufacturing process precision of nanoresonators becomes critical, as any small deviation of the optimal dimensions would result in a non-negligible shift of the resonance. On the contrary, LRI big nanospheres exhibited resonances with HWHM of the same order of magnitude as the ones exhibited by small ones but with peak values almost doubled. The peak value continued increasing for bigger radii and for this reason it has been already proposed SiO_2_ microstructures for SERS [78]. The main advantage of microstructures is the easy manufacturing process compared to nanostructures. So in general terms, dielectric nanomaterials are promising candidates for many UV spectroscopic techniques where high NFE and low heating are required. By choosing suitable dielectric material with adequate nanostructure geometry and size, low or even suppressed heating effects and also high NFE values may be achieved at molecule’s UV-absorption energies.

### 4.2. Chirality

Many biological samples exhibit chirality, a symmetry property of matter that rules how the sample interacts with light according to its polarization. A chiral molecule is characterized because its two enantiomers cannot be superimposed by means of translation and/or rotation operations. The pair of non-superimposable image molecules have many identical physical properties (molar mass, boiling point, reactivity with achiral substances, etc...). The difference between enantiomers shows up when they react with other chiral substances or with polarized light. These features are the basis of the two main pathways to detect the presence of enantiomers and/or to separate them: by either chemical reactions [154] or spectroscopic measurements [155]. The biological activity of many living organisms is selective to enantiomers. For example, The presence of the wrong enantiomer in a pharmaceutical drug can give rise to harmful side effects [154]. This is the main importance of dominating the enantiomer separation and the enantiomer concentration sensing (chiral sensing). A powerful analysis tool is the spectroscopic measurement of either light linear polarization rotation or circular polarization absorption. When linearly polarized light passes through a chiral substance, the polarization state rotates with propagation (optical activity). One enantiomer rotates the polarization in one direction an the other in the opposite direction. Another way of interaction of polarized light with chiral matter is absorption. An enantiomer has different absorption bands for right and left circularly polarized light, so for each enantiomer a CD signal can be measured, i.e., absorption difference between right and left-handed circularly polarized light. The CD spectrum of each enantiomer is in principle complementary. When the same amount of each enantiomer is present in the solution (racemic solution), the CD signal is 0 at all wavelengths as so it is the polarization rotation angle of a linear polarization. The absorption difference between enantiomers can also be used for enantiomeric purification of solutions. Strong absorption ionizes or even breaks one enantiomer present in the solution while the other remains almost unaltered for being a weaker absorber for a given wavelength and CPL handness [155]. These molecular changes in one enantiomer leads to the appearance of physical differences between the two enantiomers that can be exploited for separation.

The CD signal generated by a chiral molecule is proportional to the OCD on its surrounding. In the same sense as the near-field intensity, the OCD can be enhanced by means of light interaction with nanostructures. For circularly polarized incident light, a nonchiral nanostructure can generate OCD hot spots, as long as it simultaneously excites electric and magnetic type resonances. This enhanced OCD depends on the position where is evaluated, and again, only the values near the surface of the NP are of interest. The OCD averaged on the nanosphere surface and normalized to the incident OCD of the circularly polarized light ( $C_{CPL}$ , see Equation (6)), is defined as 〈C〉 and this is the parameter that will be discussed from now on.

**Figure 5:**
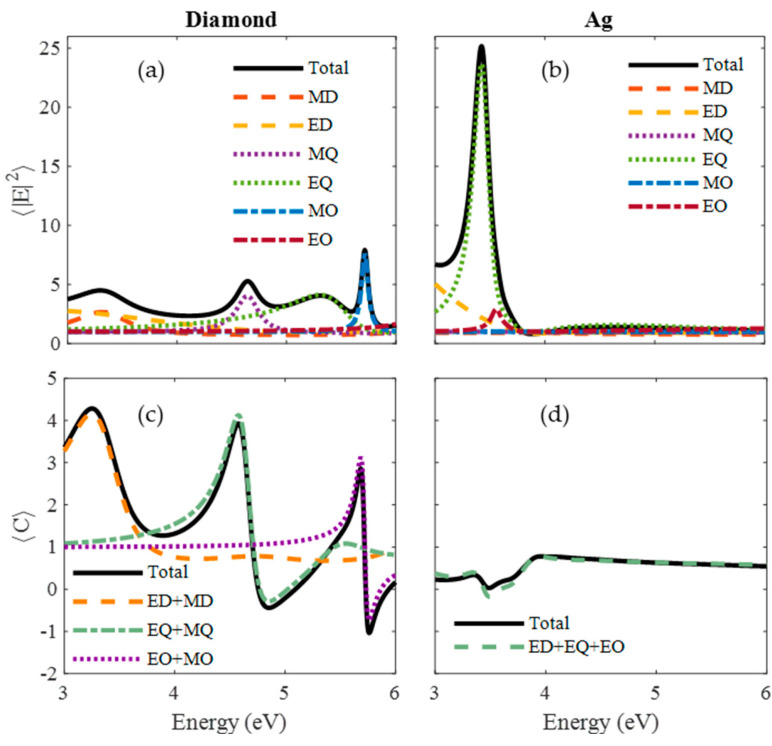
Surface-averaged near-field enhancement (NFE) and optical chirality density (OCD) enhancement spectra (black solid lines) for (**a**,**b**) diamond and (**c**,**d**) Ag. The contribution of the different multipolar terms is shown with dashed lines (ED = Electric Dipolar, MD = Magnetic Dipolar, EQ = Electric Quadrupolar, MQ = Magnetic Quadrupolar, EO = Electric Octopolar (Hexapolar), MO = Magnetic Octopolar (Hexapolar)).

In Figure 5 the $\langle |E{|}^{2}\rangle$ spectrum compared to that of 〈C〉 is shown for diamond and Ag. Diamond was chosen because it has the highest refractive index ( $\epsilon_{1}∼6$ ) of the fully transparent materials from 3 to 6 eV ( $\epsilon_{2}<<0.5$ ). The Ag nanosphere was chosen for having its Fröhlich energy in the near UV (3.5 eV) and being one of the most representative plasmonic nanomaterials. The first evident observation is that the spectral behaviours of 〈C〉 and $\langle |E{|}^{2}\rangle$ were completely different. It can be noticed from the Ag NFE spectrum that high field intensity was not a sufficient condition for enhancing OCD. The NFE resonances of diamond corresponded to the resonances of OCD, but for Ag there was no OCD resonance. If the NFE spectra of diamond and Ag nanoparticles were compared in detail, another important key factor can be highlighted: for metals, only electric resonances were generated while for HRI dielectrics, not only electric but also magnetic ones were excited. For diamond, it is interesting to point out that its first two resonances in the NFE spectrum, which corresponded to almost equal contributions of electric and magnetic resonances, were directly correlated with the most intense maxima observed in the OCD spectrum. However, for the Ag spectra, negligible contributions of magnetic resonances could be seen. This led to non-enhanced values of the OCD (values less than unity in absolute value). In fact, for diamond, the absolute maximum of NFE did not correspond to the absolute maximum of OCD enhancement since in this case, the magnetic octopole contributed much more than the electric one. The design of non-chiral nanostructures for OCD enhancement relies on the overlapping between electric and magnetic resonances, which in some nanostructures such as cylinders could be separately tuned by means of their aspect ratio [47].

**Figure 6:**
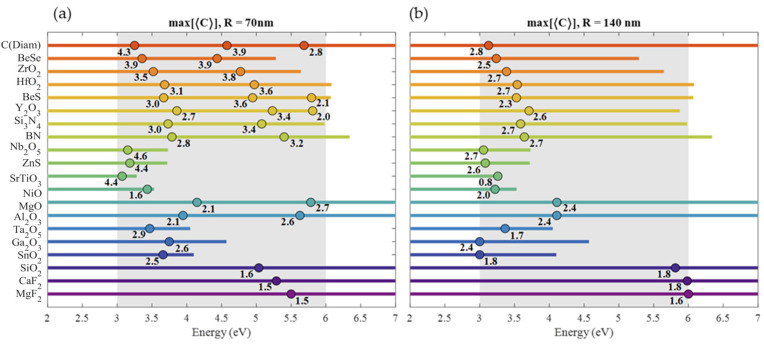
Spectral maximum of surface-averaged OCD enhancement for 70 nm (**a**) and 140 nm (**b**) radius nanospheres.

In Figure 6, a summary of the maxima of 〈C〉 for each material when nanostructured in spherical shape for both R=70 and 140 nm is shown. Numbers correspond to the values of the 〈C〉 maxima, while a dot marks their energy (only resonances between 3 a 6 eV are considered). The spectral range where $\epsilon_{2}<0.5$ is also indicated with an horizontal line. For R=70 nm, those materials with enough transparency and HRI, can exhibit up to a maximum of three resonances just like diamond in Figure 5, directly related to the fact that they exhibited three magnetic resonances: dipole, quadrupole and octopole overlapped to the tails of their counterpart electric resonances. From left to right, the first resonance arose from the mixture of ED and MD resonance, the second one from the mixture of EQ and MQ and finally, the third one from MO and EO, being the latter always less intense than the other two because of their poor overlapping given the narrowness of the octopole resonances. With increasing radius, it has to be noticed that 〈C〉 values did not increase significantly, at least not for HRI with the exception of diamond and Y_2_O_3_. It has to be considered that for these two materials, as shown in Figure 4, extraordinary high values of NFE were also observed for these same radii. These values were so high that compensate the lack of overlapping between magnetic and electric resonances. However, the 〈C〉 maximum value obtained was not significant compared to that of $\langle |E{|}^{2}\rangle$ (see Figure 4). Therefore, in terms of Kuhn’s dissymmetry factor (see Equation (5)) the absorption was strongly dominated by ordinary absorption, leading to poor enantioespecific absorption. Moreover, the resonance width for these resonances was extremely low, making tuning the resonance difficult. Unlike for HRI materials, 〈C〉 for moderate and low refractive index (MRI and LRI) increased significantly. In fact, this increment continued for LRI materials (i.e., SiO_2_, MgF_2_ and CaF_2_) until particle sizes were of the order of 600 nm, without exhibiting high and narrow $\langle |E{|}^{2}\rangle$ resonances.

**Figure 7:**
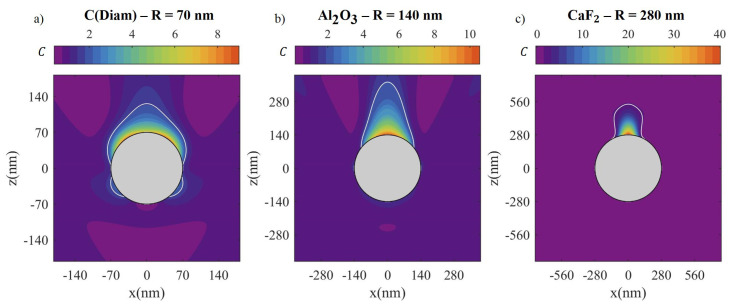
Spatial distribution of the OCD in the proximity of a spherical NP with a (**a**) 70 nm radius of diamond at 4.6 eV, (**b**) 140 nm radius of Al_2_O_3_ at 4.1 eV and (**c**) 280 nm radius of CaF_2_ at 5.7 eV. With a white line, the section where 〈C〉>2 is indicated.

So far, only surface averaged values of *C* have been discussed. However, the spatial distribution of this magnitude is especially relevant, not only for the study of the OCD enhancement distribution, but also for the *C* sign distribution. For the latter, a homogeneous sign is desired so the enantioespecific absorption contributes equally all over the nanostructure surface. The spatial response of HRI (e.g., diamond), MRI (e.g., Al_2_O_3_) and LRI (e.g., CaF_2_), is compared using an optimal radius for each material: 70 nm for diamond ( n≈2.6 ), 140 nm for Al_2_O_3_ ( n≈1.8 ) and 280 nm for CaF_2_ ( n≈1.5 ). For each material and radius, the photon energy exciting the maximum of the 〈C〉 spectrum is chosen, considering only the range 3 to 6 eV. The resulting spatial distribution is shown in Figure 7. The transversal direction to the wave propagation (z positive) has been arbitrarily chosen as the x-axis, but due to the azimutal symmetry of the distribution, any other transversal direction can be considered obtaining the same pattern. Some additional numerical data concerning the spatial distribution of *C* in the surface of the sphere is given in Table 2. For the three chosen materials, a similar optimum 〈C〉 is obtained, but the spatial distribution is very different. This is related to the fact that in each case the radius and refractive index are different. For a HRI and small radius NP, a more homogeneously distributed OCD enhancement is generated. Nanospheres of large radius and relatively LRI tend to focus the OCD on the top of the sphere just acting as a focusing lens. As a consequence, while the peak values are larger, specially for the lower refractive index material (CaF_2_), the effective area of enhanced interaction ( C>1 ) corresponds to a smaller fraction of the total. However, in absolute terms, the effective area is larger because of the larger size of the sphere. One can check that the case of HRI particles with large radius (diamond with 140 nm radius, for example) exhibits even higher peak values than LRI particles, but in this case, the sign distribution is no longer homogeneous, just as it has been already thoroughly discussed in ref. [43] for HRI particles in the IR. If the reader is interested in the spectral response obtained for large radius spheres with the materials studied here we have included some examples in the Supplementary Information.

## 5. Conclusions

We have performed an exhaustive comparative survey of the absorption, near field enhancement (NFE) and optical chirality density (OCD) properties of 20 nanostructured low-loss dielectrics in order to highlight the most promising materials for surface enhanced UV light-matter interaction. To get this goal, we have thoroughly evaluated two different magnitudes: the NFE and OCD averaged over the surface of a nanosphere with different sizes. The materials under study include oxides, nitrides, halides and chalcogenides that share the feature of having a transparency window in the UV that suppresses resistive losses (i.e., heat generation) in the NP. For this comparative study, we have classified the proposed materials attending to their refractive index.

Those materials classified as high refractive index (HRI, i.e., n> 2) present the highest NFE $\langle |E{|}^{2}\rangle$ , making them appealing for surface-enhanced spectroscopies. However, the narrowness of their resonance peaks may hamper its tuning with the light source and the probe molecule absorption bands, specially when dealing with high order resonances. As the refractive index of the nanoparticle decreases (MRI and LRI), $\langle |E{|}^{2}\rangle$ drops but the resonance peaks broaden, making the aforementioned tuning easier. Therefore, optimal conditions for surface-enhanced spectroscopies in the UV with low-loss dielectrics entails a trade off between $\langle |E{|}^{2}\rangle$ and peak width. This trade off is heavily affected by the size of the NP, specially for HRI dielectrics, since increasing the size of the NP produces an increment of $\langle |E{|}^{2}\rangle$ and a narrowing of the peak widths proportional to the refractive index of the NP.

Concerning the OCD, again HRI materials show the largest values. However, the differences between 〈C〉 values in HRI, MRI and LRI materials are lower than for $\langle |E{|}^{2}\rangle$ . Nevertheless, the spectral evolution of the peak widths is the same. In terms of the Kuhn’s disymmetry factor, the absorption is dominated by the ordinary absorption term for HRI materials, caused by their unbalanced values of $\langle |E{|}^{2}\rangle$ and 〈C〉 , specially with increasing NPs’ sizes. MRI and LRI dielectrics show more balanced values of $\langle |E{|}^{2}\rangle$ and 〈C〉 . The sign distribution of *C* around the NP, another important parameter to consider in chiral sensing, have been proven to be highly dependent on the order of the excited multipoles. Low order resonances produces *C* distributions with homogeneous sign, which is the desired experimental condition. For HRI NPs, this situation is reached with NP sizes below 200 nm. Low order resonances with uniform sign distribution of *C*, for a HRI NP red-shift out of the UV spectral range by increasing size above 200 nm. MRI and LRI dielectric NPs have larger size upper limits in order to meet the aforementioned condition, e.g., 280 nm for Al_2_O_3_ (MRI) NPs or 560 nm for CaF_2_ (LRI) NPs.

Although the values of the NFE for the evaluated dielectric NP are in general lower than those reported for metallic NP, it is important to note that achiral metal nanomaterial cannot generate OCD hot spots because of their weak magnetic LSPRs. Moreover, due to the low losses of the studied dielectric materials, both OCD and near-field hot spots are produced with no heat generation (i.e., no Joule losses), a detrimental effect that can affect both the sample and the nanostructure.

The road map presented here may prove helpful for designing dielectric nanostructures for specific applications such as surface-enhanced spectroscopies or chiral sensing in the UV. The list of materials proposed here consists of materials with well reported properties and applications so we believe that when nanostructured, their use for surface enhanced techniques is quite feasible. It is in this context that this report aims to aid researchers working towards all-dielectric UV photonics in the design of nanostructures with both NFE and OCD performance “a la carte”.

## Figures and Tables

**Table 1:** Summary of materials, their primary applications or potential applications and reported nanostructures based on those materials.

Material	Applications	Lithography	Nanoparticles (Colloidal)
MgF_2_	BOD(UV-IR), CS [71,72]	-	-
CaF_2_	BOD(UV-IR), CS [71,73]	[74]	[75]
SiO_2_	CS, BOD(VIS-IR), MOSFET, FO, SES [59,71,76,77,78]	[79,80,81]	[82]
Al_2_O_3_	CS, MRAM, ISFET [83,84,85]	[74,86]	-
MgO	CS, MRAM [83,87]	[74]	[88]
SnO_2_	n-TCF, GS, Pcat, UV-PD [89,90,91,92,93]	[91]	[94]
Ga_2_O_3_	MOSFET, SBD, HPD, n-TCF, UV-PD, Pcat, GS [95,96,97,98,99]	[100]	[101]
BN	HT-HPD, HT-UV-PD [102]	[103]	[104]
Y_2_O_3_	SS-LASER, Pcat, GS, HT-CS [51,105,106,107]	[108]	[109]
Si_3_N_4_	SES, CS, Pcat, [6,110,111]	[112,113]	-
Ta_2_O_5_	DRAM, MOSFET, ISFET, CS, Pcat [114,115,116]	[117,118]	[116]
HfO_2_	MOSFET, RRAM [119]	[120]	[121]
BeS	Blue and green LED [57]	-	-
ZrO_2_	DRAM, Pcat [122,123]	[118,124]	[125]
NiO	Pcat, p-TCF, UV-PD, GS, Battery anode, DSSC-electroanode [126,127,128,129,130,131]	-	[132]
C(Diam)	OQC, LumD, HT-HPD [68,133,134]	[68,135]	[67]
BeSe	Blue and green LED [57]	-	-
Nb_2_O_5_	DSSC-electroanode, Pcat [136,137,138]	[118]	[138]
ZnS	Pcat, p and n-TCF, LumD, UV-PD, SES [139,140,141]	[140,142]	[143]
SrTiO_3_	Pcat, GS [144,145]	-	[99]

**Table 2:** Numerical information concerning sphere surface distribution of OCD enhancement.

Material	R (nm)	$E_{\max(C)}$ (eV)	⟨C⟩	$A_{\mathrm{tot}}$ (μm^2^)	A(C>⟨C⟩) (μm^2^)	A(C>1) (μm^2^)	A(C<0) (μm^2^)
C(Diam)	70	4.58	3.9	0.062	0.021 (33%)	0.060 (97%)	0
Al_2_O_3_	140	4.1	2.4	0.246	0.046 (19%)	0.246 (100%)	0
CaF_2_	280	5.7	1.6	0.985	0.097 (10%)	0.409 (41%)	0

## Supplementary Materials

The following are available online at www.mdpi.com/xxx/s1.

## Abbreviations

The following abbreviations are used in this manuscript:

NFE	Near-field enhancement
OCD	Optical chirality density
HRI	High refractive index
MRI	Medium refractive index
LRI	Low refractive index
BOD	Bulk optical devices
CS	Coatings and substrates
FO	Fiber Optics
DRAM	Dynamic random access memories
RRAM	Resistive random access memories
MRAM	Magnetoresistive random access memories
ISFET	Ion sensitive field effect transistor
TCF	Transparent conductive films
GS	Gas sensors
Pcat	Photocatalysis
HT	High temperatures
HPD	High power devices
PD	Photodetectors
MOSFET	Metal-oxide-semiconductor field effect transistor
SBF	Schottky barrier diodes
LED	Light-emitting diode
DSSC	Dye Synthesize Solar Cells
WBS	Wide Bandgap Semiconductor
LumD	Luminescent devices
OQC	Optical quantum computing
MD	Magnetic dipole
MQ	Magnetic quadrupole
MO	Magnetic octopole
ED	Electric dipole
EQ	Electric quadrupole
EO	Electric octopole
