# Correction: Rosales et al. Non-Absorbing Dielectric Materials for Surface-Enhanced Spectroscopies and Chiral Sensing in the UV. *Nanomaterials* 2020, *10*, 2078

**DOI:** 10.3390/nano14020236

**Published:** 2024-01-22

**Authors:** Saúl A. Rosales, Francisco González, Fernando Moreno, Yael Gutierrez

**Affiliations:** 1 Department of Applied Physics, University of Cantabria, Avda. Los Castros, s/n., 39005 Santander, Spain; rosalessa@unican.es (S.A.R.); gonzaleff@unican.es (F.G.); 2 Institute of Nanotechnology, CNR-NANOTEC, Via Orabona 4, 70126 Bari, Italy

In the published study [1], a mistake was reported in the method of obtaining the mean value over the spherical nanoparticle surface of the near-field enhancement (NFE, $\langle {\left|E\right|}^{2}\rangle$) and the optical chirality density (OCD, $\langle C\rangle$). Recently, we have been advised that the way we obtained the mean values is incorrect: The mean value of the dispersion of points is obtained from the evaluation of the OCD and NFE using the coordinates generated on the surface of the sphere from a homogeneous 2D mesh grid in the polar angles (θ,ϕ). This generates an accumulation of points in the poles of the sphere, resulting in a higher weight for values on the poles with respect to the values on the equator.

All the presented corrections concern the following figures in the requested article, Figure 3, Figure 4a,b, Figure 5, Figure 6, Figures S4a,c and S5a,c, as well as Table 2 that includes information obtained from Figure 7. The corrections consist of calculating again the values shown in those figures and table, according to a correct set of points, that avoids the overweighting of values in the poles. The picture below shows the distribution of N ≈ 1000 points employed to obtain the spherical surface-averaged values of the NFE and OCD in the original article (a) vs. distribution for approximately the same number of points employed in this correction (b).


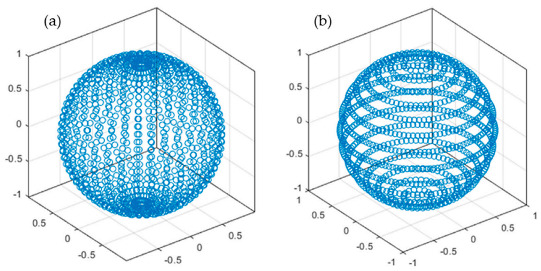


## Text Correction

There was an error in the original publication. No information about the procedures of obtaining the surface average was indicated.

A correction has been made to **Sections 4 (Results)** and *4.1. Near-Field Enhancement and Absorption Efficiency*, paragraph 2:

“When achiral and isotropic samples are analyzed, the light absorption is only affected by the molecular polarizability and field intensity. So, in this case, a high field intensity is desired to enhance the molecular absorption and thus obtain more detailed matter information [149,150]. This is the basis of many surface-enhanced spectroscopic techniques like SERS [149,150], SEIRA [150], and SEF [150,151]. Because the electric near-field intensity, ${\left|E\right|}^{2}$, varies with position in the nanoparticle surface, we average this magnitude over the sphere’s surface, denoting the average as $\langle {\left|E\right|}^{2}\rangle$. To perform such an average, a set of equi-distributed points must be generated on the surface of the nanoparticles. For spherical geometries, this is not straightforward for an arbitrary number of points. Here, the set of points for evaluating the mean of ${\left|E\right|}^{2}$ is carried out by following the procedures explained in [152]. Of course, that average will be normalized to the incident electric field intensity ${\left|E_{0}\right|}^{2}$. Apart from the peak values of $\langle {\left|E\right|}^{2}\rangle$, the half-width at half-maximum (HWHM) of the $\langle {\left|E\right|}^{2}\rangle$ resonances and the absorption efficiency $Q_{abs}$ at the energy of the maximum $\langle {\left|E\right|}^{2}\rangle$ are magnitudes to be considered. Large values of $Q_{abs}$ result in high absorption rates and thus a non-negligible heating of both the sample and nanostructure, which can result in detrimental changes in their properties [2]. To avoid this, low $Q_{abs}$ values are desirable in the $\langle {\left|E\right|}^{2}\rangle$ resonance energy. On the other hand, small HWHMs (high Q-factors) are desired for enhancing detection limits. For exploiting the best yield of high Q-factors resonance, an almost perfect tuning between the light source, the nanostructure resonance, and the molecule’s natural frequency is critical. This tuning is not always trivial to fulfill. Consequently, depending on the experimental conditions (light source, manufacturing precision, molecule’s absorption band, etc.), high Q-factors are important or unnecessary. In this section, we analyze these parameters and their relationship for the materials considered in this research.”

## Error in Figure 3

In the original publication, there was a mistake in Figure 3 as published. The presented values of $\langle {\left|E\right|}^{2}\rangle$ are slightly incorrect due to the wrong distribution of the points on the surface of the nanoparticle. The corrected Figure 3 appears below.

**Figure 3:**
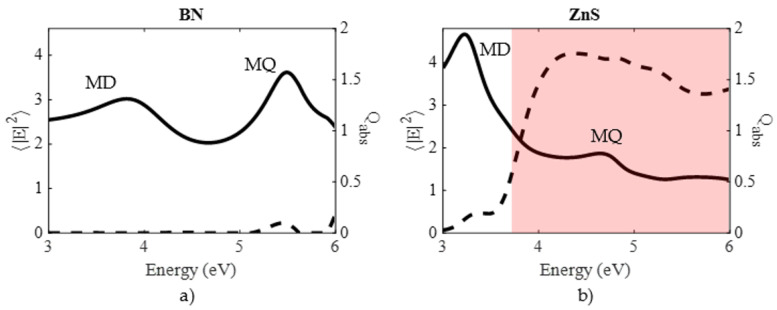
Surface-averaged electric field intensity (solid line, left axis) and absorption efficiency (dashed line, right axis) for (**a**) BN and (**b**) ZnS nanospheres of 70 nm radius. The red shaded area indicates the range where $\varepsilon_{2}>0.5$.

## Error in Figure 4

In the original publication, there was a mistake in Figure 4a,b as published. The presented values of $\langle {\left|E\right|}^{2}\rangle$ are incorrect due to the wrong distribution of the points on the surface of the nanoparticle. The corrected Figure 4a,b appear below. 

**Figure 4:**
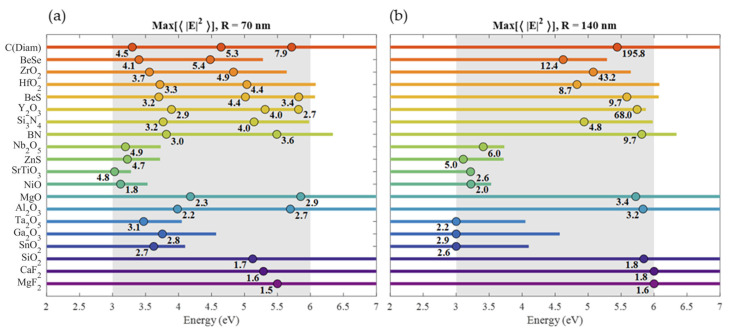
Summary of the $\left\langle {\left|E\right|}^{2}\right\rangle$ maxima values in the [3, 6] eV range for (**a**) 70 nm and (**b**) 140 nm radius spheres. For 70 nm radius, only magnetic resonances are indicated, while for 140 nm radius, only the most intense resonance is indicated. The dots indicate the energies of the maxima, while the line length indicates the range of energies where $\varepsilon_{2}<0.5$. Under each dot, the maximum value of $\left\langle {\left|E\right|}^{2}\right\rangle$ is indicated.

## Error in Figure 5

In the original publication, there was a mistake in Figure 5 as published. The presented values of $\langle {\left|E\right|}^{2}\rangle$ and $\langle C\rangle$ are slightly incorrect due to the wrong distribution of the points on the surface of the nanoparticle. The corrected Figure 5 appears below. 

**Figure 5:**
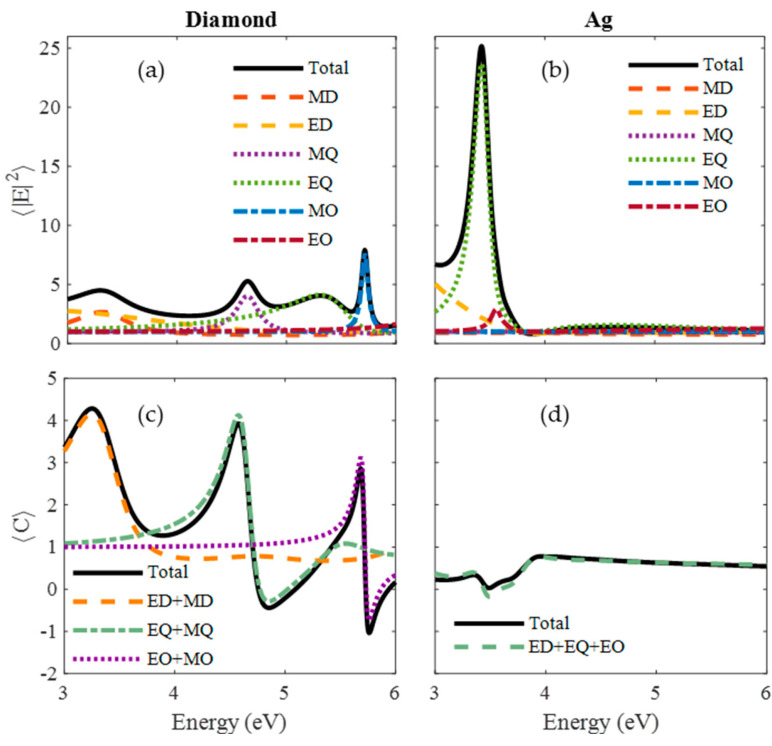
Surface-averaged near-field enhancement (NFE) and optical chirality density (OCD) enhancement spectra (black solid lines) for (**a**,**b**) diamond and (**c**,**d**) Ag. The contribution of the different multipolar terms is shown with dashed lines (ED = Electric Dipolar, MD = Magnetic Dipolar, EQ = Electric Quadrupolar, MQ = Magnetic Quadrupolar, EO = Electric Octopolar (Hexapolar), MO = Magnetic Octopolar (Hexapolar)).

## Error in Figure 6

In the original publication, there was a mistake in Figure 6 as published. The presented values of $\langle C\rangle$ are incorrect due to the wrong distribution of the points on the surface of the nanoparticle. The corrected Figure 6 appears below. 

**Figure 6:**
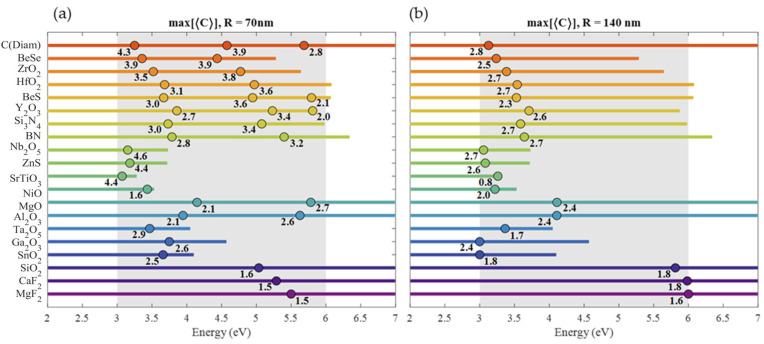
Spectral maximum of surface-averaged OCD enhancement for 70 nm (**a**) and 140 nm (**b**) radius nanospheres.

## Error in Table 2

In the original publication, there was a mistake in Table 2 as published. The presented values of $\langle C\rangle$ and area where $C>\langle C\rangle$ are incorrect due to the wrong distribution of the points on the surface of the nanoparticle. The corrected Table 2 appears below. 

**Table 2:** Numerical information concerning sphere surface distribution of OCD enhancement.

Material	R (nm)	$E_{max(C)}$ (eV)	⟨C⟩	$A_{tot}$ (μm^2^)	$A(C>\left\langle C\right\rangle )$ (μm^2^)	A(C>1) (μm^2^)	A(C<0) (μm^2^)
C(Diam)	70	4.58	3.9	0.062	0.021 (33%)	0.060 (97%)	0
Al_2_O_3_	140	4.1	2.4	0.246	0.046 (19%)	0.246 (100%)	0
CaF_2_	280	5.7	1.6	0.985	0.097 (10%)	0.409 (41%)	0

## Error in Figure S4a,c

In the original publication, there was a mistake in Figure S4a,c as published. The presented values of $\langle {\left|E\right|}^{2}\rangle$ are incorrect due to the wrong distribution of the points on the surface of the nanoparticle.

## Error in Figure S5a,c

In the original publication, there was a mistake in Figure S5a,c as published. The presented values of $\langle C\rangle$ are incorrect due to the wrong distribution of the points on the surface of the nanoparticle. 

## Reference Correction

New added ref. 152: Beltrán, C.; Etayo, U. The diamond ensemble: A constructive set of spherical points with small logarithmic energy. *J. Complex.* **2020**, *59*, 101471.

## Conclusions

In the conclusions, no special mention is given to the extremely high values of surface means. In fact, high-order resonances are advertised as challenging to exploit due to their spectral narrowness and unbalanced OCD values with respect to NFE. These properties are not affected by the recalculation of the means. All the calculations were carried out with the same method, so the conclusions concerning comparisons between HRI, MRI, and LRI are still valid. In other words, with this correction, NFE and OCD values are affected in the same way for all the materials.

The authors apologize for any inconvenience caused and state that the scientific conclusions are unaffected. This correction was approved by the Academic Editor. The original publication has also been updated.

## References

[1] Rosales S.A., González F., Moreno F., Gutiérrez Y. (2020). Non-Absorbing Dielectric Materials for Surface-Enhanced Spectroscopies and Chiral Sensing in the UV. Nanomaterials.

