# Supplementary material for: Non-Absorbing Dielectric Materials for Surface-Enhanced Spectroscopies and Chiral Sensing in the UV
Source: Nanomaterials (Basel). 2020 Oct 21;10(10):0. doi: 10.3390/nano10102078 (PMC7589615; doi:10.3390/nano10102078)
Supplement: Supplementary file 1 [file nanomaterials-10-02078-s001.pdf]

nanomaterials MDPI

Supplementary Materials

Non-Absorbing Dielectric Materials for Surface-Enhanced Spectroscopies and Chiral Sensing in the UV

Saúl A. Rosales [1], Francisco González [1], Fernando Moreno [1,*] and Yael Gutierrez [2,*]

[1] Department of Applied Physics, University of Cantabria, Avda. Los Castros, s/n., 39005 Santander, Spain; rosalessa@unican.es (S.A.R.); gonzaleff@unican.es (F.G.)
[2] Institute of Nanotechnology, CNR-NANOTEC, Via Orabona 4, 70126 Bari, Italy
* Correspondence: ; morenof@unican.es (F.M.); gutierrezyael@uniovi.es (Y.G.)

S.1. Optical constants

Partially UV-transparent materials

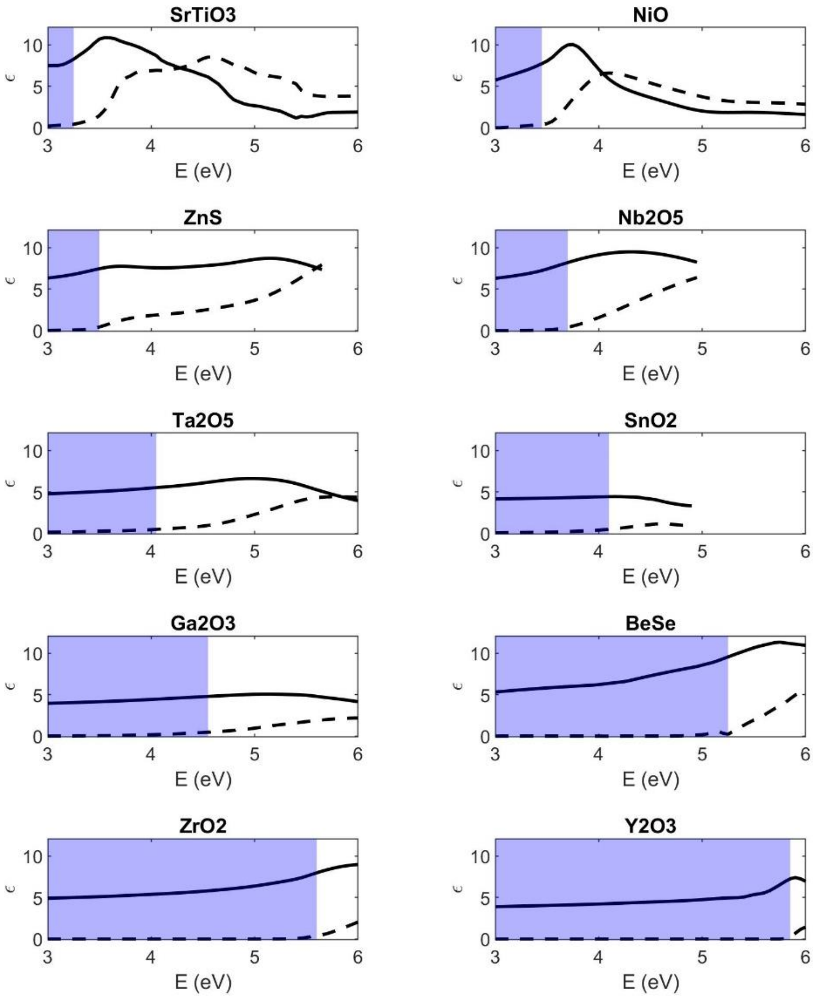

Figure S1. Real (solid line) and imaginary (dashed line) parts of the permittivity as a function of energy. Only partially UV-transparent materials are shown. The shaded area indicates the range of energies for which the imaginary part stays below 0.5.

Fully UV-transparent materials

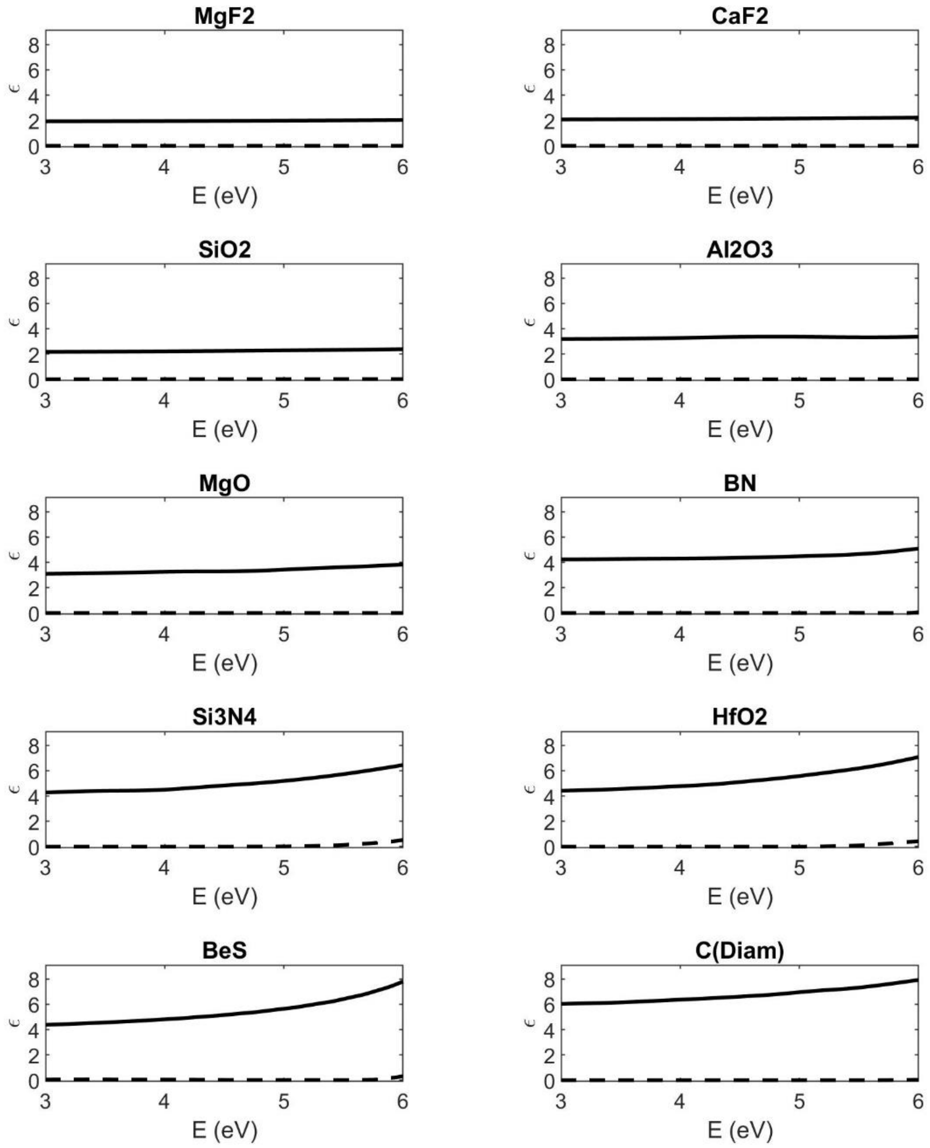

Figure S2. Real (solid line) and imaginary (dashed line) part of the permittivity as a function of energy. Only fully UV-transparent materials are considered, i.e. they have an imaginary part of the permittivity lower than 0.5.

S.2. Transparency criteria

The electromagnetic intensity I decays exponentially in a media with complex refractive index $\hat{n} = n + ik$ as

$$I = I_0 e^{-z/l_d} \tag{1}$$

where the decay length, l_d is

$$l_d = \lambda/4\pi k \tag{2}$$

and the imaginary part of the refractive index, k, is a function of the real and imaginary parts of the permittivity, ε_1 and ε_2 respectively.

$$k = \sqrt{\frac{1}{2}\sqrt{\varepsilon_1^2 + \varepsilon_2^2} - \varepsilon_2} \quad (3)$$

Figure S3 shows the decay length as a function of the complex permittivity at a wavelength λ = 380 nm. At that wavelength, all the materials exhibit an $\varepsilon_2 < 0.5$. Only three materials, i.e., NiO, Ta_2O_5 and $SrTiO_3$, show an $\varepsilon_2 > 0.2$ at that wavelength, as indicated by the dots. The rest of them have even smaller ε_2 and thus even bigger decay lengths. Two dashed lines indicate the pair of values $(\varepsilon_1, \varepsilon_2)$ that give the constants values of decay length 140 nm (white dashed) and 280 nm (black dashed), which are the two diameters considered for the nanospheres at this work. The condition $\varepsilon_2 < 0.5$ is enough restrictive to ensure a sufficiently high transparency, i.e. a decay length longer than the diameters of the spheres. In the list considered, only materials with $\varepsilon_1 > 4$ may exhibit an absorption band in the range 3-6 eV. As we can see from figure S3, if $\varepsilon_1 > 4$ and $\varepsilon_2 > 0.5$, the decay length is no longer superior to 280 nm so the resonances in spheres of this diameter become overdamped. Materials with $\varepsilon_1 < 4$ have their absorption bands at energies above 8 eV so the exhibit neglectable values for ε_2 in the range from 3-6 eV, i.e. the decay length is orders of magnitudes larger than the diameters of the spheres.

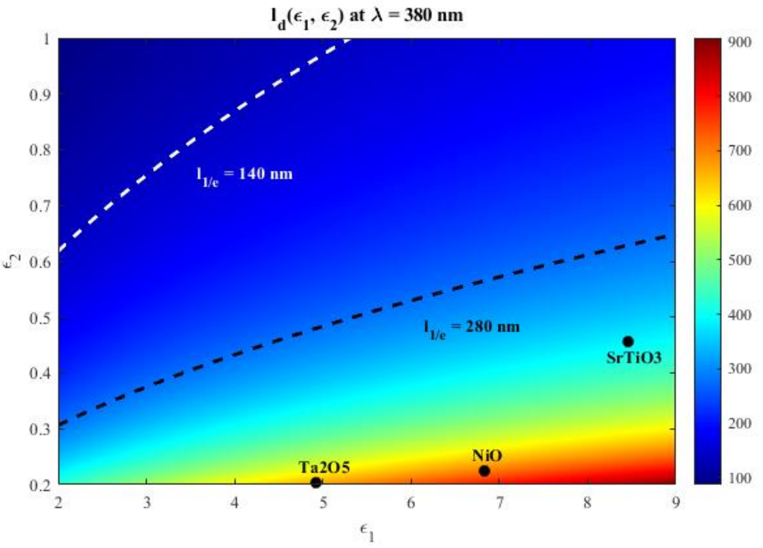

Figure S3. Decay length as a function of real and imaginary part of the permittivity.

S.3. Suprawavelength nanoparticles for NFE and OCD enhancement

In general terms, the NFE becomes greater with bigger nanoparticles with consequent spatial confinement of the electromagnetic field and an increase of the Q-factor of the resonances. This triple effect is more dramatic for HRI nanoparticles than for MRI or LRI one. As an example of this, we show in Figure S4 the spectral distribution of the NFE averaged over the surface of the sphere (a,c) and the spatial distribution over the surface of the sphere when the diameter is 280 nm and the incident wavelength is inferior to 230 nm (b,d). The considered materials are Diamond (a,b) and Al_2O_3 (c,d) surrounded by air.

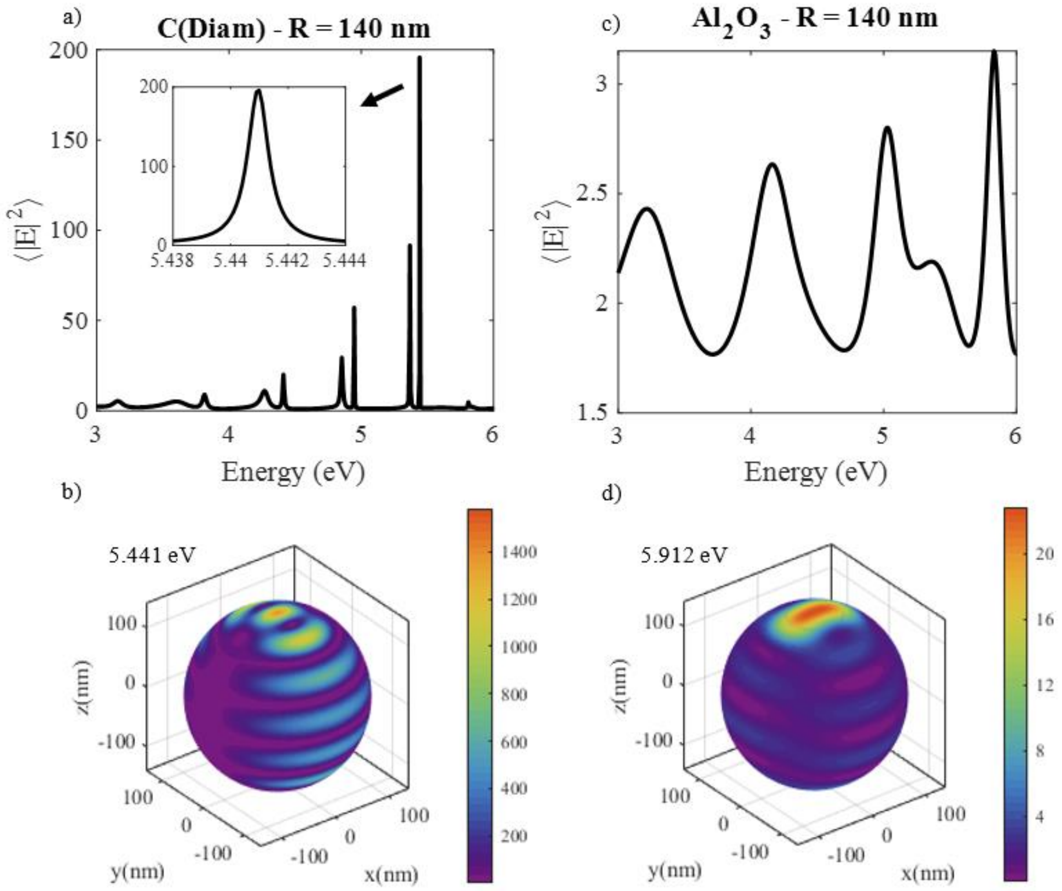

Figure S4. Surface averaged NFE as a function of energy, and spatial distribution of the NFE over a 140 nm radius sphere of (a-b) diamond and (c-d) Al_2O_3 nanoparticles.

The tendency with the radius is not that simple for the OCD enhancement. The OCD is large if the NFE is so, but more importantly if the magnetic type (H-type) and the electric type (E-type) resonances overlap. Magnetic and electric modes overlap more for smaller refractive index nanoparticles. As well, the lower order resonances that appear for smaller radius at a given wavelength, because of their lower Q-factors, tend to overlap more. So, in principle, for HRI spheres the radius must be small enough to achieve moderate Q-factors, enhancing the overlapping between E-type and H-type resonances. This is illustrated in figure S4: for diamond (HRI), when the sphere has a size of 280 nm, the resonances are completely resolved while for Al_2O_3 (MRI) the resonances appear much more overlapped. For LRI and MRI, the radius may be increased much more than for HRI, in order to achieve more intense resonances without significantly losing the overlapping between E-type and H-type modes, as the later ones exhibit moderate Q-factor even for big radius as discussed in the article.

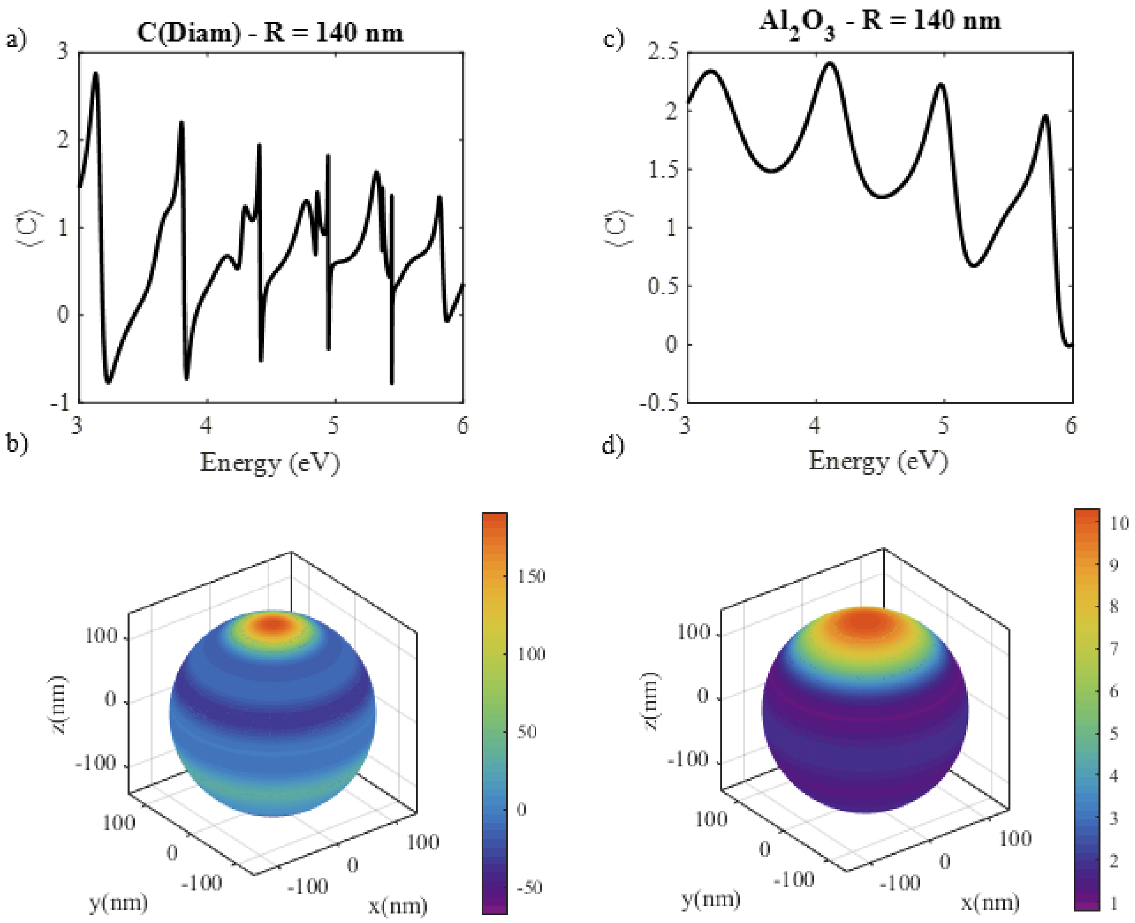

Figure S5. Surface averaged OCD as a function of energy, and spatial distribution of the OCD over a 140 nm radius sphere of (a-b) diamond and (c-d) Al_2O_3 nanoparticles.

As we can see in Figure S5, when the diameter is big enough, in LRI and MRI spheres it is possible to achieve relatively high and sign uniform OCD resonances that may be easily tuned because the resonances are wide. On the contrary, for HRI spheres with excessively big radius turns up in spectrally narrow resonances of OCD. Moreover, this OCD values are neglectable compared to the NFE and the spatial sign distribution is not uniform. For this reason, small HRI particles can be of the same utility as big LRI and MRI particles, at least when they are used surrounded by air.
